# *OsCOP1* regulates embryo development and flavonoid biosynthesis in rice (*Oryza sativa* L.)

**DOI:** 10.1007/s00122-021-03844-9

**Published:** 2021-05-05

**Authors:** Backki Kim, Rihua Piao, Gileung Lee, Eunbyeol Koh, Yunjoo Lee, Sunmin Woo, Reflinur, Wenzhu Jiang, Endang M. Septiningsih, Michael J. Thomson, Hee-Jong Koh

**Affiliations:** 1 Department of Agriculture, Forestry and Bioresources, Research Institute for Agriculture and Life Sciences, and Plant Genomics and Breeding Institute, Seoul National University, Seoul, 08826 Republic of Korea; 2 Rice Research Institute, Jilin Academy of Agricultural Sciences, Gongzhuling, Jilin, 136100 China; 3 College of Pharmacy and Research Institute of Pharmaceutical Science, Seoul National University, Seoul, 08826 Republic of Korea; 4 Indonesian Center for Agricultural Biotechnology and Genetic Resources Research and Development, IAARD, Bogor, 16111 Indonesia; 5 Jilin Province Engineering Laboratory of Plant Genetic Improvement, College of Plant Science, Jilin University, Changchun, 130062 China; 6 Department of Soil and Crop Sciences, Texas A&M University, College Station, TX 77483 USA

## Abstract

**Key message:**

Novel mutations of *OsCOP1* were identified to be responsible for yellowish pericarp and embryo lethal phenotype, which revealed that OsCOP1 plays a crucial role in flavonoid biosynthesis and embryogenesis in rice seed.

**Abstract:**

Successful production of viable seeds is a major component of plant life cycles, and seed development is a complex, highly regulated process that affects characteristics such as seed viability and color. In this study, three yellowish-pericarp embryo lethal (*yel*) mutants, *yel*-*hc*, *yel*-*sk*, and *yel*-*cc*, were produced from three different *japonica* cultivars of rice (*Oryza sativa* L). Mutant seeds had yellowish pericarps and exhibited embryonic lethality, with significantly reduced grain size and weight. Morphological aberrations were apparent by 5 days after pollination, with abnormal embryo development and increased flavonoid accumulation observed in the *yel* mutants. Genetic analysis and mapping revealed that the phenotype of the three *yel* mutants was controlled by a single recessive gene, *LOC*_*Os02g53140*, an ortholog of *Arabidopsis thaliana CONSTITUTIVE PHOTOMORPHOGENIC 1* (*COP1*). The *yel*-*hc*, *yel*-*sk*, and *yel*-*cc* mutants carried mutations in the RING finger, coiled-coil, and WD40 repeat domains, respectively, of *OsCOP1*. CRISPR/Cas9-targeted mutagenesis was used to knock out *OsCOP1* by targeting its functional domains, and transgenic seed displayed the *yel* mutant phenotype. Overexpression of *OsCOP1* in a homozygous *yel-hc* mutant background restored pericarp color, and the aberrant flavonoid accumulation observed in *yel-hc* mutant was significantly reduced in the embryo and endosperm. These results demonstrate that *OsCOP1* is associated with embryo development and flavonoid biosynthesis in rice grains. This study will facilitate a better understanding of the functional roles of *OsCOP1* involved in early embryogenesis and flavonoid biosynthesis in rice seeds.

**Supplementary Information:**

The online version contains supplementary material available at 10.1007/s00122-021-03844-9.

## Introduction

Light is a crucial signaling component that controls a variety of physiological and developmental processes in morphogenesis, germination, and flowering in plants. Light signaling is also involved in metabolic alterations, including the biosynthesis of chlorophyll (Ilag et al. 1994; Pattanayak et al. 2005) and several types of pigments (Takos et al. 2006; von Lintig et al. 1997; Zoratti et al. 2014). These light signaling pathways are triggered upon perception of light signals by multiple photoreceptors, such as red/far red light-sensing phytochromes (phyA to phyE), UV-A/blue light-sensing cryptochromes (cry1 and cry2), phototropins (phot1 and phot2), Zeitlupes (ZTL), and the UV-B-sensing photoreceptor (UVR8), which transmit signals to genes that regulate developmental and metabolic processes such as anthocyanin biosynthesis in higher plants (Jiao et al. 2007; Kami et al. 2010).

The light signaling regulator CONSTITUTIVELY PHOTOMORPHOGENIC 1 (COP1) is an essential negative regulator of light-mediated plant development. In darkness, COP1 acts as an E3 ubiquitin ligase to repress light signaling by targeting numerous photomorphogenic-promoting transcription factors, including ELONGATED HYPOCOTYL5 (HY5), HY5-HOMOLOG (HYH), LONG AFTER FAR-RED1 (LAF1), and LONG HYPOCOTYL IN FAR-RED1 (HFR1), for ubiquitination and degradation in the nucleus (Holm et al. 2002; Osterlund et al. 2000; Seo et al. 2003; Yang et al. 2005). In addition to acting as a repressor of photomorphogenesis (Ang et al. 1998; Deng et al. 1991; Ma et al. 2002; Seo et al. 2003), COP1 functions as a crucial regulator in several biological processes, such as flowering time (Jang et al. 2015; Tanaka et al. 2011; Xu et al. 2016a), circadian regulation (Yu et al. 2008), stomatal development (Kang et al. 2009; Lee et al. 2017), UV-B signaling (Favory et al. 2009; Huang et al. 2014; Yin et al. 2015), abiotic stress responses (Kim et al. 2016), and hormone signaling (Pacin et al. 2016; Zheng et al. 2017). In particular, COP1 plays a central role in regulating anthocyanin biosynthesis and fruit coloration by complexing with other proteins (Li et al. 2012; Liang et al. 2020; Maier et al. 2013).

The COP1 protein comprises three distinct structural domains that are conserved in both higher plants and vertebrates: a zinc-binding RING finger motif, a coiled-coil domain, and a WD40 repeat domain (Stacey et al. 1999). These highly modulated domains of COP1 are involved in self-association, dimerization, and protein interactions with other proteins (Holm et al. 2001; Torii et al. 1998). The RING finger domain plays a critical role in mediating the recognition of substrates as well as the transfer of ubiquitin to substrates and to the RING finger domain itself (Joazeiro and Weissman 2000; Kosarev et al. 2002). The coiled-coil domain is involved in dimerization and interactions with other proteins, including CIP1, SPA1, and CSU2 (Hoecker and Quail 2001; Lee et al. 2018; Matsui et al. 1995; Xu et al. 2015). In COP1, the WD40 domain is found at the C-terminus and consists of seven WD40 repeats that mediate protein–protein interactions. COP1 directly interacts with several regulators, such as HY5 and HYH, and photoreceptors (cytochromes and UVR8) via its WD40 domain (Holm et al. 2001, 2002; Ponnu et al. 2019; Yin et al. 2015).

Mutagenesis is an important strategy to create mutations which are valuable sources for functional genomics as well as crop improvement. In particular, relatively small genome size of rice has enabled a genome-wide saturation mutagenesis and revelation of full spectrum of gene functions by generating allelic series of mutations with small population (Henikoff and Comai 2003). Over the past several decades, numerous mutagenesis methods have been developed for rice, but chemical and physical mutagens have been widely applied to induce genetic variability and to study gene function due to its simple and low cost strategy (Viana et al. 2019; Wang et al. 2013). The genome changes between wild-type and mutants caused by chemical and physical mutagen have been identified efficiently in recent years using Targeting Induced Local Lesions IN Genomes (TILLING) (Till et al. 2007) and MutMap (Abe et al. 2012), next-generation sequencing (NGS)-based gene mapping tool in rice. These researches through mutagenesis would be helpful to identify unknown pathways or genes and could be directly applied to the rice breeding.

Genome editing technology using the Clustered Regularly Interspaced Short Palindromic Repeats/CRISPR-associated Cas9 (CRISPR/Cas9) system is a powerful and promising tool for precise modification of genome sequences in crop plants. In recent years, the CRISPR/Cas9 system has been widely adopted for crop improvement due to its simplicity, specificity, efficiency, and feasibility compared with zinc finger nuclease and transcription activator-like effector nuclease editing technologies (Khan 2019). Studies employing CRISPR/Cas9 genome editing tools have been used to validate gene functions and improve useful traits in rice, such as herbicide resistance (Li et al. 2016a; Sun et al. 2016), rice blast disease resistance (Wang et al. 2016), cold tolerance (Zeng et al. 2019), salt tolerance (Zhang et al. 2019), amylose content (Sun et al. 2017), and grain size and/or yield (Li et al. 2016b; Xu et al. 2016b). Ongoing improvements in genome editing tools will accelerate the pace of crop improvement and gene validation experiments.

The function of *COP1* in photomorphogenic development and anthocyanin biosynthesis has been extensively studied in *Arabidopsis*. However, little is known regarding the role of *COP1* in flavonoid biosynthesis and embryo development in rice. In this study, novel *OsCOP1* mutants that exhibited a yellowish pericarp color and seed lethality were identified, and CRISPR/Cas9 gene editing tool was used to validate the mutant phenotype. These findings provide new insights into the regulation of flavonoid biosynthesis and embryo development in rice.

## Materials and methods

### Plant materials and growth conditions

Three yellowish-pericarp embryo lethal (*yel*) mutants, *yel-hc*, *yel-sk*, and *yel-cc*, were derived from *japonica* rice cultivars Hwacheong, Samkwang, and Chucheong, respectively, by induction with *N*-methyl-*N*-nitrosourea. Due to their embryonic lethality, the mutants were maintained as heterozygotes. F_2_ mapping populations were developed from crosses between the three heterozygous *yel* mutants and Milyang 23 (M.23, Korean *indica*-type rice). F_2_ populations derived from crosses between the three heterozygous *yel* mutants and their respective wild types, Hwacheong, Samkwang, and Chucheong, were used to calculate segregation ratios. F_2_ populations, wild-type varieties, and *yel* mutants were cultivated in a paddy field at the Experimental Farm of Seoul National University, Suwon, Korea.

### Phenotypic analysis

Dimensions of dehulled rice including length, width, and thickness were measured in a total of 90 matured grains (30 seeds × 3 replicates) using digimatic calipers (Mitutoyo, Japan). Hundred-grain weights were measured (100 seeds × 3 replicates; 10% water content) using an analytical balance (CAS Corporation, NJ, USA). Phenotypic data collected from the three *yel* mutants and their respective wild types were statistically analyzed using SAS version 9.4 (SAS Institute Inc., Cary, NC, USA).

### Morphological and histological observations of developing seed

Caryopses of Hwacheong and the *yel*-*hc* mutant were collected at several stages of seed development and imaged using HD’MEASURE software (HANA Vision Systems, Korea). For histological examination, samples were collected at 3 days after pollination (DAP), 4 DAP, 5 DAP, 7 DAP, 10 DAP, and 40 DAP (matured), and fixed in formalin-acetic acid-alcohol fixative (3.7% formaldehyde, 5% acetic acid, and 50% ethanol) at 4 °C. Seeds were dehydrated by soaking in a series of ethanol solutions of increasing concentration (50%, 70%, 80%, 90%, 95%, and 100%) for 3 h each, followed by soaking in 100% ethanol overnight at room temperature. The dehydrated samples were soaked in a series of mixtures of absolute ethanol and Histo-Clear (3:1, 1:1, 1:3) for 3 h each, followed by soaking in 100% Histo-Clear overnight at room temperature. For paraffin infiltration, Paraplast® (Sigma, USA) was gradually added to Histo-Clear (3:1, 1:1, 1:3) at 60 °C. Finally, samples were stored in 100% melted paraffin for 24 h at 60 °C. Paraffin-infiltrated samples were embedded in an embed block and cut into 10-μm sections with an HM 340 E Rotary Microtome (Microm Lab, Germany). The sections were stained with 1% Safranin O solution and observed under a CX31 Microscope (Olympus, Japan).

### Map-based cloning of *yel* gene

Genomic DNAs were extracted from a total of 898 matured mutant-type seeds and young heterozygous and wild-type leaves of F_2_ individuals derived from the *yel*-*hc* x Milyang23 population. To determine the locus of the gene underlying the *yel*-*hc* mutant, bulked segregant analysis (BSA) (Michelmore et al. 1991) was performed with the first batch of STS sequence-tagged-site (STS) markers, which were developed by designing primers based on the DNA sequence differences between *indica* and *japonica* rice cultivars (Chin et al. 2007). Next, additional STS primers were designed with Primer3 version 0.4.0 (http://frodo.wi.mit.edu/primer3) based on available rice genome sequence data (http://www.ncbi.nlm.nih.gov) and were used to refine the region of the *yel-hc* locus. Additionally, a total of 192 F_2_ individuals were used for each of the *yel*-*sk* and *yel*-*cc* mutant gene mapping. The overall mapping strategy and primers were the same as for *yel*-*hc*. Primers designed and used in this study are detailed in Table S1.

### Sequence analysis of candidate genes

The full-length genomic DNA sequence of candidate genes in Hwacheong and *yel*-*hc* was divided into several overlapping segments and then amplified. PCR products were purified using a PCR purification kit (iNtRON Biotechnology, Korea), TA-cloned into the pGEM-T Easy Vector (Promega, USA), and transformed into *E*. *coli* strain DH5α. The obtained sequences were compared using CodonCode Aligner software (version 1.6.3; CodonCode Corporation, MA, USA).

### Vector constructs and rice transformation

CRISPR/Cas9 vectors to knock out *COP1* were constructed as described previously (Lowder et al. 2015). Briefly, two guide RNAs (gRNAs) for each target were designed using the web-based tools CRISPR RGEN Tools (http://www.rgenome.net/cas-designer/) (Park et al. 2015) and CRISPRdirect (https://crispr.dbcls.jp/) (Naito et al. 2015). The two individual gRNAs were cloned into two gRNA expression vectors, pYPQ131C (Addgene plasmid #69,284) and pYPQ132C (Addgene plasmid #69,285), under the expression of the OsU6 promoter. The gRNA expression cassettes were then assembled into the Golden Gate recipient vector pYPQ142 (Addgene plasmid #69,294). For the Cas9 entry vector, pYPQ165 (Addgene #109,327), which is driven by an egg cell-specific promoter, was used. Final binary T-DNA vectors were prepared using a Gateway assembly LR reaction with the Cas9 entry vector (pYPQ165), gRNA cassettes (pYPQ142), and pMDC99 (binary vector). The full-length Os*COP1* cDNA was amplified from Hwacheong cDNA and used for construction of the overexpression vector. The amplified fragment was transferred into the pMDC32 Gateway binary vector containing the cauliflower mosaic virus (CaMV) 35S promoter using a pCR™ 8/GW/TOPO® TA Cloning Kit (Invitrogen, USA) and Gateway™ LR Clonase™ II Enzyme mix (Invitrogen). The resulting *OsCOP1* cDNA overexpression construct was denoted as 35S::*OsCOP1*. For the promoter-GUS (*β*-glucuronidase) assay, the genomic sequence containing the putative promoter region (− 2215 to − 1 bp upstream of the start codon) of *OsCOP1* was amplified from genomic DNA and cloned into the binary vector pHGWFS7 using Gateway® BP and LR clonase enzyme mixes (Invitrogen).

The final constructs were transformed into seeds of the *japonica* cultivar Dongjin, via *Agrobacterium*-mediated transformation using the LBA4404 strain, as described previously (Nishimura et al. 2006), with slight modifications. Primers used for vector construction and genotyping are listed in Table S1.

### Histochemical GUS assay

Histochemical GUS-staining was performed as described previously (Jefferson et al. 1987). X-Gluc buffer solution was vacuum-infiltrated into tissue samples, which were incubated overnight at 37 °C, after which the staining solution was serially replaced with 95% and 70% (w/v) ethanol to remove the chlorophyll. Staining patterns in tissue samples were examined using HD’MEASURE software (HANA Vision, Korea).

### Quantitative real-time PCR analysis

Total RNA was extracted from 7-day-old seeds of the three *yel* mutants and their respective wild types, 35S::*OsCOP1*, knockout lines, and Dongjin using a TaKaRa MiniBEST Plant RNA Extraction Kit (Takara Bio, Japan). Total RNA samples were converted into first-strand cDNA using M-MLV reverse transcriptase (Promega, USA), and qRT-PCR was performed using SYBR Premix Ex Taq (Takara Bio, Japan) on a CFX96™ Real-Time PCR Detection System (Bio-Rad, USA), according to the manufacturer’s instructions. Primers used for qRT-PCR analysis are listed in Table S1. Expression levels of *OsCOP1* were normalized relative to *ACTIN*, a housekeeping gene. Data were analyzed using the comparative Ct method. Expression levels were compared using a two-tailed Student’s *t* test.

### Quantitative analysis of major flavonoids using HPLC

HPLC sample preparation and quantitative analysis were performed as described previously (Kim et al. 2018). Whole grain, embryo, and endosperm tissues from *yel*-*hc*, Hwacheong, and transgenic overexpression seeds were powdered, and three technical replicates were prepared for quantitative analysis of six representative flavonoid compounds (isoorientin, isoorientin 2″-*O*-glucoside, vitexin 2″-*O*-glucoside, isoscoparin 2″-*O*-glucoside, isoscoparin, and isovitexin). HPLC was performed on a XBridge C18 column (250 × 4.6 mm, 5 μm; Waters, Milford, MA, USA) using an Ultimate 3000 UHPLC system (Thermo Fisher Scientific, Inc., MA, USA) equipped with an autosampler, a gradient system, and a diode array detector (DAD). Chromatograms were acquired at 365 nm, and photodiode array spectra were recorded from 190 to 800 nm. The injection volume of each sample was 5 μL.

## Results

### Morphological characterization of *yel* mutants

**Fig. 1:**
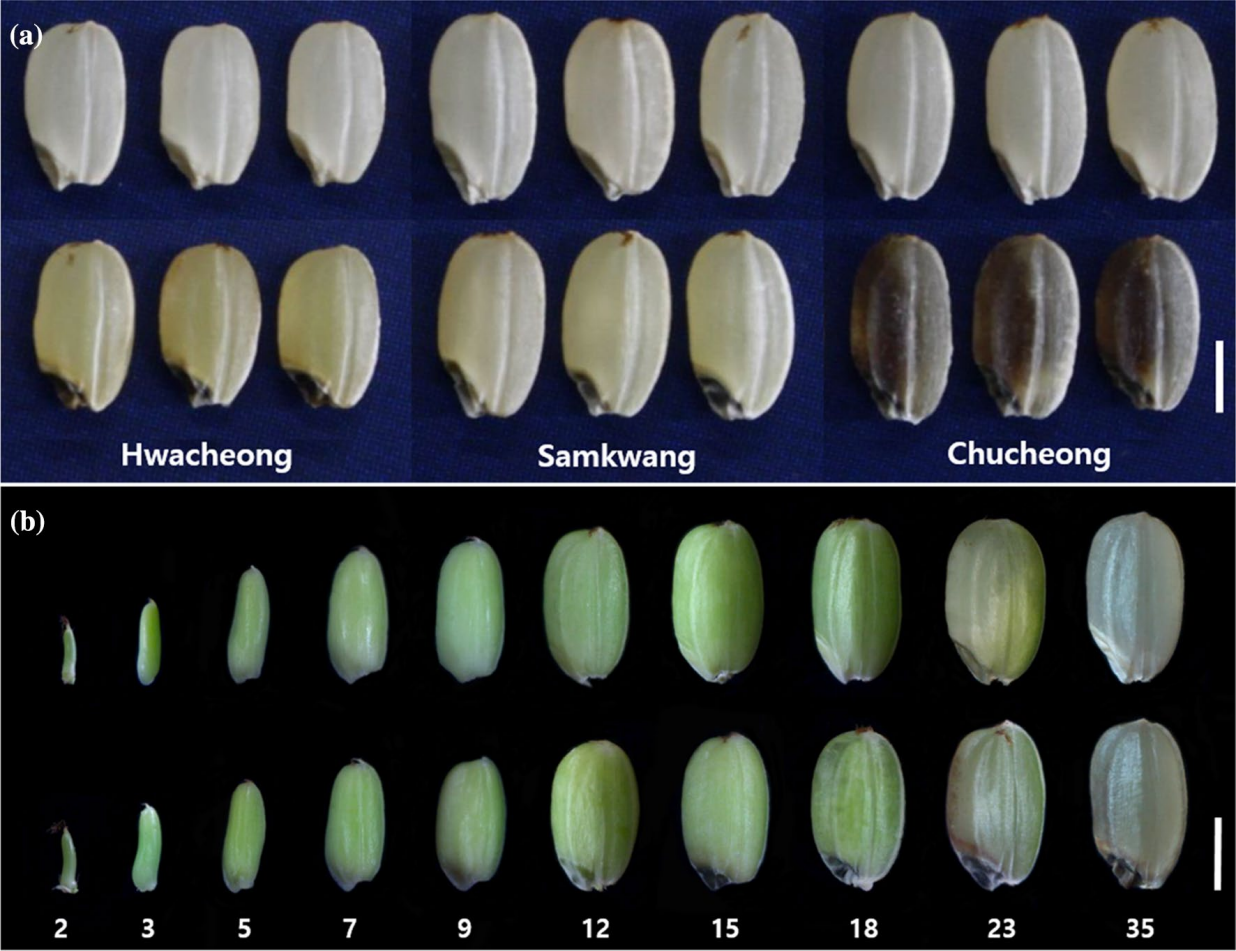
Comparison of grain morphology and development between wild-type and *yel* mutant. **a** Phenotype of wild-type and *yel* mutant seeds. Upper and lower panels represent wild-type and *yel* mutants, respectively. **b** Morphological differences during seed development between wild-type (Hwacheong) and *yel*-*hc* mutant. Upper and lower panels show seed development of wild-type (Hwacheong) and *yel*-*hc* mutant, respectively. Numbers at the bottom of the subpanel indicate days after pollination. Scale bar = 2 mm

Three yellowish-pericarp embryo lethal (*yel*) mutants, *yel-hc*, *yel-sk*, and *yel-cc*, were derived from *japonica* rice cultivars Hwacheong, Samkwang, and Chucheong, respectively. The most prominent characteristic of the mutants was a bicolor grain phenotype. Embryos were black in all three *yel* mutants, and the pericarp was yellowish in *yel*-*hc* and *yel*-*sk* grains and mixed yellow-purple in *yel*-*cc* grains (Fig. 1a). The black pigmentation in *yel* mutant embryos was first observed at approximately 5 DAP and the yellowish pericarp color was clearly visible at 35 DAP, the late-maturing stage (Fig. 1b). All three *yel* mutants exhibited significantly reduced grain length and thickness compared with wild type, and grain width was also significantly lower in the *yel-sk* and *yel-cc* mutants. Correspondingly, hundred-grain-weight was also significantly lower in *yel* mutants than in wild type. However, there was no significant difference between *yel* mutants and wild type in the length-to-width ratio of de-hulled rice grains (Table 1). These results indicated that the *yel* mutations influenced embryo and pericarp color, grain size, and grain weight in rice.

**Table 1:** Characteristics of grain size and weight in *yel* mutants and wild-type seeds

Trait	Length (mm)	Width (mm)	L/W ratio	Thickness (mm)	Hundred-grain weight (g)
WT-*hc*	4.65 ± 0.035	2.80 ± 0.059	1.66 ± 0.035	1.96 ± 0.049	1.71 ± 0.015
*yel-hc*	4.56 ± 0.066**	2.78 ± 0.064	1.64 ± 0.038	1.89 ± 0.039**	1.62 ± 0.02**
WT-*sk*	5.01 ± 0.100	2.90 ± 0.090	1.73 ± 0.055	1.99 ± 0.068	2.06 ± 0.011
*yel-sk*	4.91 ± 0.090**	2.82 ± 0.068*	1.74 ± 0.032	1.91 ± 0.067**	1.94 ± 0.012**
WT-*cc*	5.14 ± 0.121	2.85 ± 0.081	1.80 ± 0.054	2.03 ± 0.057	2.11 ± 0.014
*yel-cc*	5.01 ± 0.083**	2.79 ± 0.073*	1.80 ± 0.036	1.88 ± 0.055**	1.86 ± 0.005**

Data are means ± SD. Student’s *t* test was used to generate p values; * and ** indicate *p* < 0.05 and *p* < 0.01, respectively

### Embryo development of *yel* mutants

**Fig. 2:**
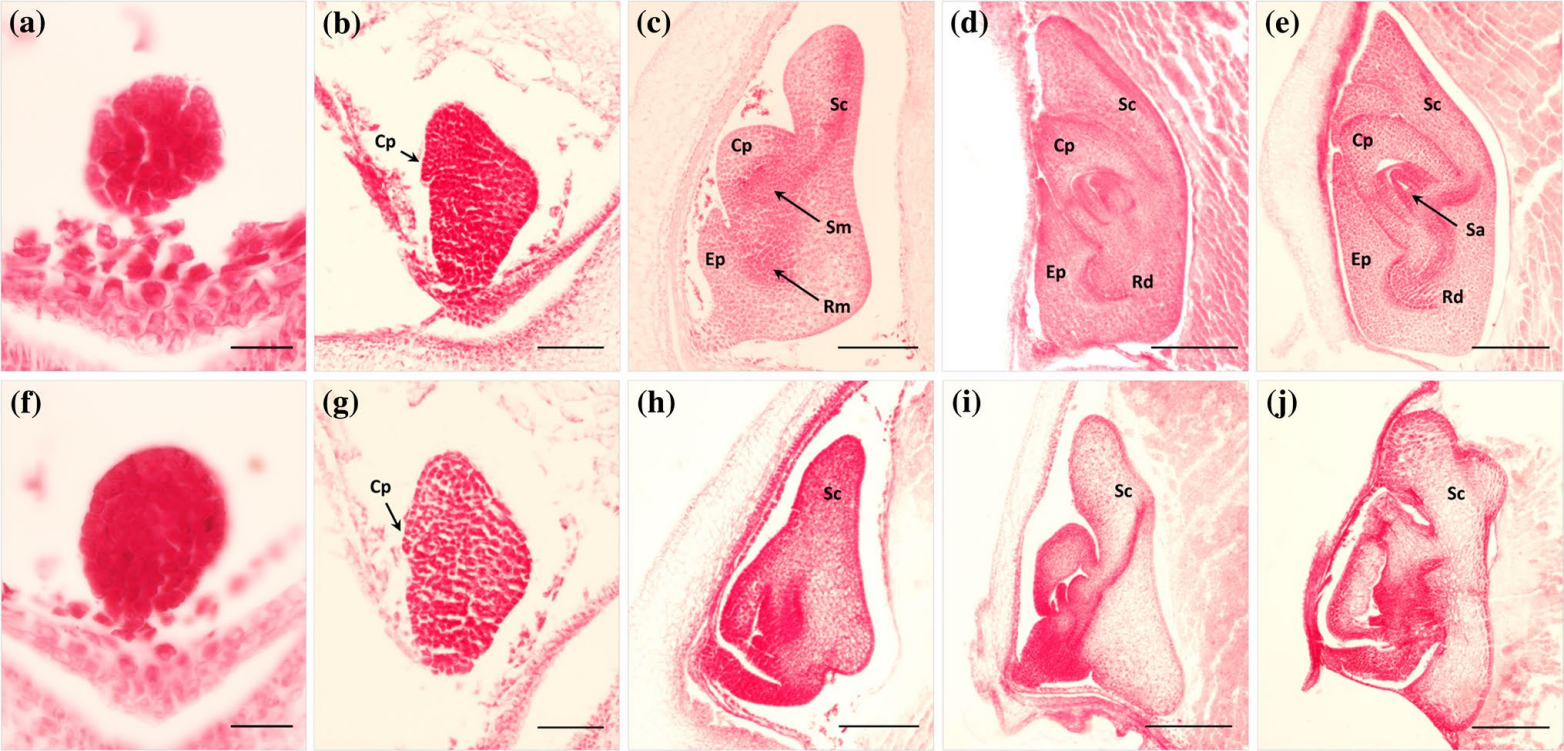
Embryo development in wild-type and *yel* mutant. Upper and lower panels represent wild-type (Hwacheong) and *yel*-*hc* embryo development, respectively. **a** and **f** Embryo at 3 DAP (bar = 20 μm), **b** and **g** 4 DAP (bar = 100 μm), **c** and **h** 5 DAP (bar = 200 μm), **d** and **i** 7 DAP (bar = 300 μm), and **e** and **j** 10 DAP (Bar = 300 μm). Cp, coleoptile; Ep, epiblast; Rd, radicle; Sc, scutellum; Sm, shoot meristem; Rm, root meristem; Sa, shoot apex

Homozygous *yel* mutant seeds failed to germinate under both standard germination testing and on MS medium (data not shown). Longitudinal sections of *yel-hc* seeds were examined to determine when defects occured during *yel* mutant embryo development*.* Normal embryonic development was observed in both *yel*-*hc* and wild-type embryos during early embryogenesis, including at the globular stage (Fig. 2a and f). The coleoptile primordium was differentiated in both the wild-type and *yel*-*hc* mutant, and no morphological differences were observed at 4 DAP (Fig. 2b and g). In wild-type embryos, the coleoptile, epiblast, and scutellum had formed, and the shoot and root meristems had begun to differentiate, at 5 DAP. However, the overall morphology of *yel-hc* embryos differed from that of wild type at 5 DAP. It was not possible to determine organ differentiation in the *yel-hc* embryo as the shape and structure of organs were unclear (Fig. 2c and h). At more advanced developmental stages, all organs were normally differentiated and developed in wild-type embryos, whereas abnormally differentiated organs were observed in *yel*-*hc* embryos (Fig. 2d, e and i, j). In the mature embryo, all of the organs were evident in wild type, but, despite completion of embryogenesis, irregular and unstructured organs were observed in the *yel*-*hc* mutant (Fig. S1). These results demonstrated that the mutation of *YEL* caused defects in embryonic differentiation and development, resulting in seed lethality and failure to germinate in *yel* mutants.

### Genetic analysis and map-based cloning of the *YEL* gene

As homozygous *yel* mutants were lethal, heterozygotes of each *yel* mutant and their respective wild type were used in crosses. The segregation ratios of wild-type and heterozygous plants for *yel*-*hc*, *yel*-*sk*, and *ye1*-*cc* in F_1_ progenies were 12:14, 11:10, and 14:11, respectively. These ratios were close to a 1:1 ratio, and the segregation ratios of wild-type and *yel* mutant grains produced by F_1_ plants fitted a 3:1 ratio (WT:*yel*-*hc* = 479:145, *χ*^2^ = 1.034 < *χ*^2^ 0.05(1) = 3.841, *p* = 0.31; WT:*yel*-*sk* = 496:154, *χ*^2^ = 0.593 < *χ*^2^ 0.05(1) = 3.841, *p* = 0.44; WT:*yel*-*cc* = 418:128, *χ*^2^ = 0.706 < *χ*^2^ 0.05(1) = 3.841, p = 0.40). These results indicated that the *yel* phenotype was controlled by a single recessive gene.

**Fig. 3:**
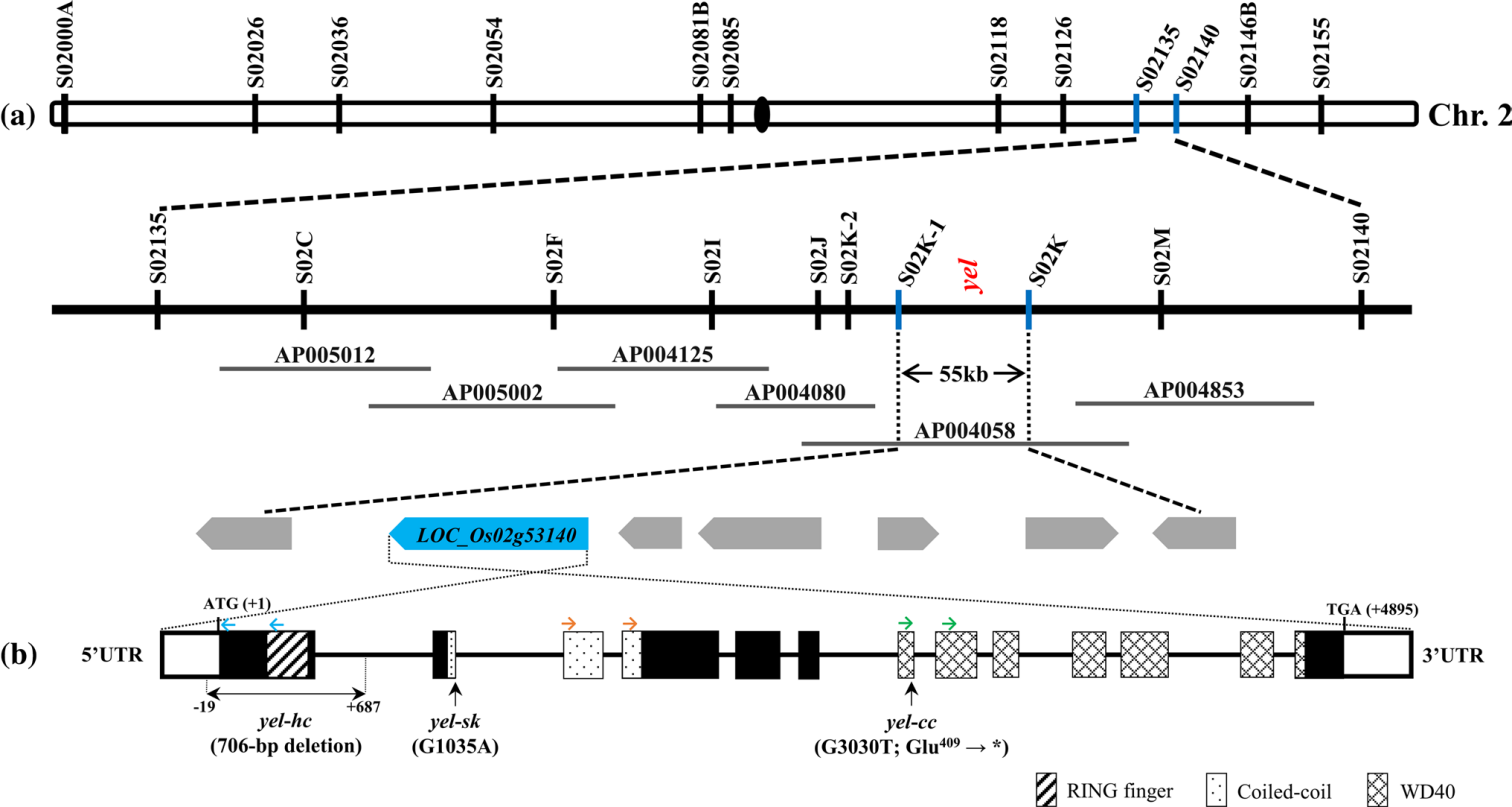
Map-based cloning of the *YEL* gene. **a** Schematic diagram of *YEL* locus mapping. **b** Gene and protein domain structure of *YEL*. Black lines, white solid boxes, and black solid boxes indicate introns, untranslated regions, and exons, respectively. Mutation positions are marked with black arrows. ATG and TGA indicate the initiation and termination codons, respectively. The position and direction of gRNAs are marked by arrows above the gene structure

An F_2_ population derived from a cross between a *yel*-*hc* heterozygous mutant and Milyang23 (M.23) was used to map the locus responsible for the *yel*-*hc* phenotype. For the first step of genetic mapping, BSA was performed using 84 polymorphic STS markers, and the *yel*-*hc* locus was localized between two flanking markers, S02135 and S02140, on chromosome 2 (Fig. 3a). To refine the flanking region, BSA was again conducted using an F_2_ population of 840 individuals with newly designed STS markers between the two flanking markers (Table S1). The *yel*-*hc* locus was finally mapped to a region of approximately 55 kb between the S02K-1 and S02K markers (Fig. 3a). Seven predicted open reading frames (ORFs) were located within this candidate region, and analysis of wild-type and *yel*-*hc* mutant sequences revealed a 706 bp deletion in the *yel*-*hc* mutant at *LOC*_*Os02g53140* (*Os02g0771100*), an ortholog of *AtCOP1* (Fig. 3b). This 706 bp deletion included part of the 5′ untranslated region (− 19 to − 1, where the A of ATG is + 1), the entire coding sequence (CDS) of exon 1 (+ 1 to + 342), and part of intron 1 (+ 343 to + 687), resulting in the loss of the conserved RING finger motif of *LOC*_*Os02g53140* (Fig. 3b). A similar mapping strategy and markers confirmed that *yel*-*sk* and *yel*-*cc* harbored mutations in the same gene as *yel*-*hc*. In the *yel*-*sk* mutant, a single base change (G to A) was discovered at the splicing donor site of the 5′ end of intron 2, within the coding region for the coiled-coil motif (Fig. 3b). In the *yel*-*cc* mutant, a single point mutation (G to T) was identified in exon 7 that generated a premature stop codon (GAG to TAG; Glu^409^ to stop) in the WD40 repeat motif (Fig. 3b). Three transcripts were identified in homozygous *yel*-*sk* seed. The first, most abundant, transcript contained the entire 473 bp of intron 2; the second included 64 bp of intron 2; and the third transcript type was the wild-type form. The insertion of the intron 2 sequence was predicted to introduce a premature termination codon at residue 145, resulting in the loss of the coiled-coil and WD40 functional motifs (Figure S2). These results indicated that the mutations identified in *LOC*_*Os02g53140* were responsible for the yellowish pericarp and embryo lethal phenotypes in *yel* mutants.

### Validation of mutations causing the *yel* phenotype

**Fig. 4:**
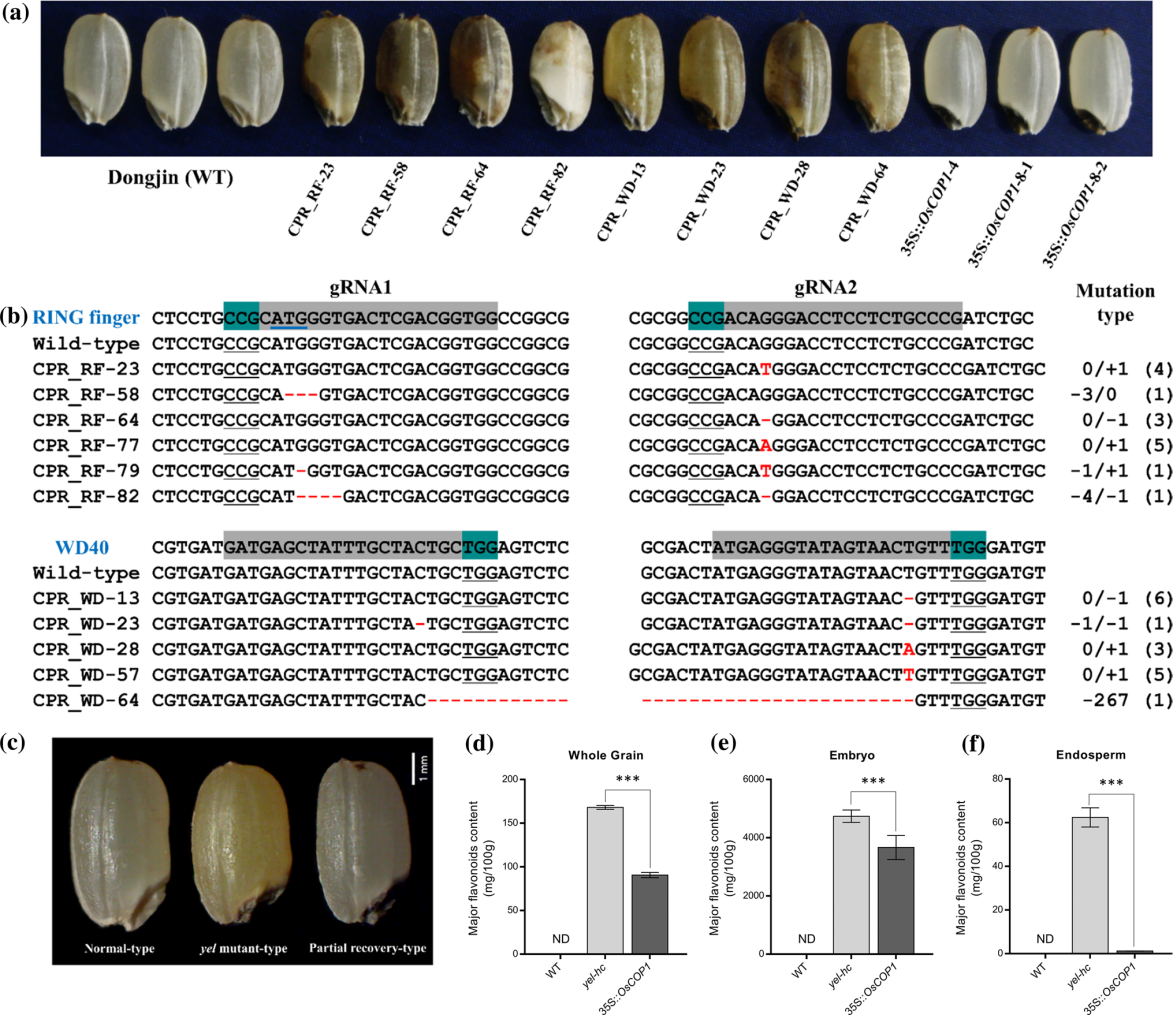
CRISPR/Cas9 mediated knockout and overexpression for complementation of *yel* mutations. **a** Grain phenotype of wild-type rice (Dongjin) and transgenic seeds. **b** Sequence comparison of target regions in *OsCOP1*. The start codon/RING finger domain and WD40 repeat domain were targeted for the *yel*-*hc* and *yel*-*cc* mutations, respectively. Red dashes and letters indicate deletions and insertions, respectively, in transgenic lines. The start codon, target sequence and protospacer adjacent motif are indicated by blue underline, gray boxes, and green boxes/black underline, respectively. Mutation types are shown to the right of each mutated sequence (−; deletion, + ; insertion). Numbers in parentheses indicate the number of mutated individuals identified from transgenic T_1_ seeds. **c** Phenotype of grains segregated from F_2_ progenies with the 35S::*OsCOP1* transgene in the homozygous *yel*-*hc* background. Normal-type (left), *yel* mutant-type (middle), and partial recovery-type (right) seeds (bar = 1 mm). **d**–**f** Contents of six major flavonoids in whole grain (**d**), embryo (**e**), and endosperm (**f**) of the wild-type (Hwacheong), *yel*-*hc*, and partial recovery-type grains (35S::*OsCOP1*). ND; not detected. Error bars represent SD for three technical experiments. Asterisks indicate statistical significance, as determined by Student’s *t* test (***p < 0.001)

To assess the functions of the individual OsCOP1 domains and the effects of the *yel* mutations, sequences for each domain were disrupted using the CRISPR/Cas9 system. Two gRNAs for each of the three constructs were designed to target crucial mutation points or domains, including the RING finger, coiled-coil, and WD40 repeat domains (Fig. 3b). An egg cell-specific Cas9 promoter was used to overcome lethality during tissue culture and increase the frequency of mutated homozygous T_1_ seeds generated from positive T_0_ transgenic plants. T_1_ seeds exhibiting the *yel* mutant phenotype were observed when the RING finger and WD40 repeat motifs of the *OsCOP1* gene were targeted (Fig. 4a), but no T_1_ seeds exhibiting the *yel* phenotype were generated upon targeting of the coiled-coil motif, even though several positive T_0_ transgenic events occurred. The mutations were confirmed by Sanger sequencing the target region of each domain in T_1_ seeds displaying the *yel* mutant phenotype. The most frequently identified mutations were single base pair insertion and deletion mutations. A large fragment deletion between two gRNAs induced in transgenic seeds targeting the WD40 repeat domain was also observed (Fig. 4b). The insertions and deletions introduced into the *OsCOP1* gene in seeds with *yel* phenotypes were all predicted to lead to frameshifts and premature stops, and thereby to produce aberrant proteins. These results demonstrated that the *yel* mutant phenotype was caused by loss-of-function of crucial domains in *OsCOP1*.

To complement the *yel* phenotype, the full-length *OsCOP1* cDNA was introduced into wild-type Dongjin rice under the control of the 35S promoter. Eight positive transgenic lines were produced and crossed with a heterozygous *yel*-*hc* mutant to introduce the overexpression construct (35S::*OsCOP1*) into the homozygous *yel*-*hc* genetic background. F_1_ plants harboring 35S::*OsCOP1* were selected, and the phenotypes of F_2_ progenies from the self-pollinated F_1_ plant were observed. As shown in Fig. 4c, three different types of seed were segregated from the F_2_ progenies: normal-type (normal pericarp and embryo color), *yel* mutant-type (yellowish-pericarp and black colored embryo), and partial recovery-type (normal pericarp color but black colored embryo). A total of 93 F_2_ seeds were genotyped to find the fully recovered seeds containing 35S::*OsCOP1* transgene in homozygous *yel*-*hc* mutant background. All 20 *yel* mutant-type seeds showed *yel*-*hc* homozygous genotype (*yel*/*yel*), and all the partial recovery-type seeds contained 35S::*OsCOP1* transgene in homozygous *yel*-*hc* mutant background (*yel*/*yel* + 35S_*OsCOP1*). However, among the normal-type seeds, none of the fully recovered transgenic *yel* seeds carrying 35S::*OsCOP1* transgene were detected. (Table S2).

In our previous study, we demonstrated that high levels of flavonoids accumulated in the embryo and endosperm of *yel*-*hc* mutant grains (Kim et al. 2018). The contents of six major flavonoids were estimated in the embryo, endosperm, and whole grain of wild-type, *yel*-*hc* and partial recovery-type seeds. The total amounts of all six flavonoids were significantly reduced by approximately 46%, 23%, and 98% in the whole grain, embryo, and endosperm of partial recovery-type (35S::*OsCOP1*) seeds, respectively, compared with the *yel*-*hc* mutant seeds (Fig. 4d–f). The lack of accumulation of flavonoids was observed in the endosperm of partial recovery-type seeds overexpressing the 35S::*OsCOP1* transgene, resulting in reduced flavonoid levels and restoration of wild type pericarp color. However, although the total amounts of the six main flavonoids were significantly reduced in the partial recovery-type seeds, high levels of flavonoids still accumulated in the embryo. In addition, partial recovery-type seeds still failed to germinate on both MS media and under standard germination conditions (data not shown). These results suggested that overexpression of the *OsCOP1* gene in *yel*-*hc* seeds partially restored the *yel* phenotype by reducing flavonoid accumulation in the embryo and pericarp.

### Relative expression of *YEL*

**Fig. 5:**
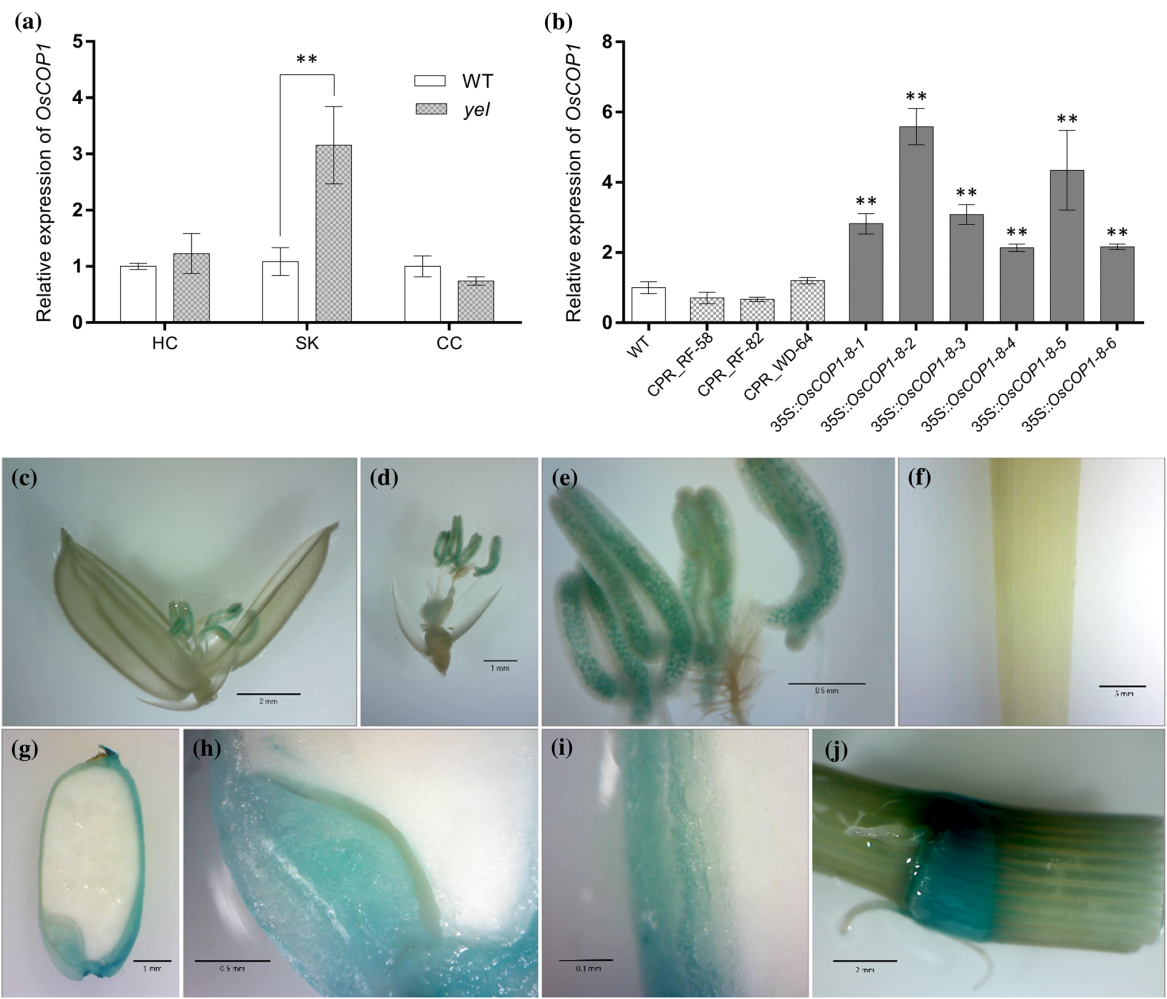
Expression patterns of the *YEL* gene. **a** Relative expression levels of *OsCOP1* in *yel* mutant and corresponding wild-type seeds using qRT-PCR. HC, Hwacheong; SK, Samkwang; CC, Chucheong. **b** Relative expression levels of *OsCOP1* in wild-type (Dongjin), knockout, and partial recovery-type (35S::*OSCOP1*) seeds. Expression levels of *OsCOP1* were normalized relative to ACTIN. Error bars represent SD for three technical experiments. Asterisks indicate statistical significance, as determined by Student’s *t* test (*p < 0.05; **p < 0.01). **c**–**j** GUS expression patterns in various organs in *OsCOP1* promoter::GUS transgenic plants. **c** Spikelet; **d**, floral organ; **e**, pollen; **f**, leaf; **g**, seed; **h**, embryo; **i**, pericarp; **j**, node

Quantitative real-time PCR (qRT-PCR) analysis using developing seeds (7 DAP) showed that expression of *OsCOP1* was significantly higher in the *yel*-*sk* mutant than in wild type, but no significant differences were observed between wild-type and *yel*-*hc* and *yel*-*cc* mutants (Fig. 5a). No statistically significant differences in relative *OsCOP1* expression level were observed between wild-type and CRISPR/Cas9 knockout seeds, which was consistent with the expression pattern of the *yel*-*hc* and *yel*-*cc* mutants. By contrast, *OsCOP1* transcript levels were significantly higher in 35S::*COP1* transgenic T_2_ seeds than in wild-type and *yel* mutant seeds or knockout lines (Fig. 5b).

### *OsCOP1* promoter–GUS expression pattern

Transgenic plants were generated containing the GUS reporter gene under the control of the *OsCOP1* promoter region, and GUS expression was observed in several organs. GUS signals were detected primarily in pollen, node, embryo, and pericarp tissues, but no expression was detected in leaf, spikelet, stigma, or endosperm tissues (Fig. 5c–j). Interestingly, GUS staining in seed was concentrated in the embryo and pericarp, consistent with the flavonoid accumulation pattern in *yel* mutant seed.

## Discussion

COP1 has been extensively studied as a repressor of plant photomorphogenesis, alongside DET (DE-ETIOLATED) and FUS (FUSCA), which also exhibit light-grown phenotypes in darkness. Many *cop/det/fus* mutants have been screened and identified in *Arabidopsis* (McNellis et al. 1994; Wei and Deng 1996), with the majority shown to cause constitutive photomorphogenesis. However, some *cop/det/fus* mutant alleles, such as *cop1-5*, *cop1*-*8*, *det1*-*6*, and *fus7*-*1*, had high levels of anthocyanin accumulation in cotyledons and leaves, and exhibited seedling lethal phenotypes under dark conditions (Castle and Meinke 1994; Ma et al. 2003; Stacey et al. 2000). Many of these phenotypically similar mutants exhibiting the ‘*fusca*’ phenotype were revealed to be allelic to each mutant group (Castle and Meinke 1994; Chory et al. 1989; Misera et al. 1994). However, unlike *Arabidopsis*, few *cop1* mutants have been reported in rice. A loss-of-function mutation in *PETER PAN SYNDROME* (*PPS*), an ortholog of Arabidopsis *COP1*, produced dwarfed dark-green plants that showed a prolonged juvenile phase and early flowering in both short-day and long-day conditions (Tanaka et al. 2011). In this study, three allelic *cop1* mutants were isolated that exhibited embryonic lethality and accumulated flavonoids in the pericarp and embryo (Figs. 1, 3). The three *cop1* mutants are likely to be complete loss-of-function mutants and, based on the severity of the phenotype, the *yel* phenotype likely represents the *cop1* null phenotype in rice. To the best of our knowledge, *cop1* null mutants exhibiting a *fusca* phenotype similar to those of *Arabidopsis* mutants have not yet been reported in rice.

Two distinctive differences were apparent between rice *yel* mutants and *Arabidopsis cop1* null mutants with the *fusca* phenotype, such as *cop1*-*5*, namely, differences in embryonic development and lethality and differences in pigmentation. First, despite germination delays, *Arabidopsis cop1* null mutant seeds completed embryogenesis and germinated successfully, although seedling growth was severely retarded and viable plants did not develop (Castle and Meinke 1994; McNellis et al. 1994; Stacey et al. 2000). In addition, despite the lethal nature of the *Arabidopsis cop1* null mutants, morphological abnormalities in embryogenesis were not observed for all the lethal alleles (McNellis et al. 1994). By contrast, in rice, all the homozygous recessive *yel* mutant seeds failed to germinate, and all showed defective embryo morphologies during embryogenesis (Fig. 2 and Fig. S1). Due to this embryonic lethality, it was impossible to observe the photomorphogenic phenotype in homozygous *yel* mutant plants. Studies on embryogenesis in plants indicate that there are crucial developmental events in early embryogenesis that are important for establishing seedling architecture after germination, such as embryo pattern formation, polarity, positioning, and cell fate determination. Although these embryogenic processes are similar among plant species, there are some differences in the patterns of cell division and organ differentiation positioning (Radoeva et al. 2019; Zhao et al. 2017). Embryogenesis in *Arabidopsis* and rice differ in their cell division patterns, position of shoot apical meristem with respect to embryo polarity, position of radicle differentiation, and the effects of embryo–endosperm interactions on embryo development (Itoh et al. 2005; Nagasawa et al. 2013). Rice and *Arabidopsis* also differ in their embryogenesis-related gene expression patterns and regulatory networks (Itoh et al. 2016). Taken together, this suggests that the differences in morphological defects between rice and *Arabidopsis* embryos in *cop1* null mutants may be due to differences in developmental processes between monocots and dicots during early embryogenesis.

Second, the seed coat and cotyledons in *Arabidopsis cop1* null mutants were dark purple as a consequence of anthocyanin accumulation (Castle and Meinke 1994; McNellis et al. 1994; Stacey et al. 2000). In rice, *yel* mutants displayed yellowish pericarps and black embryos in dried seeds. Our previous study showed that the accumulated pigments in the embryo and pericarp were mainly *C*-glycosyl flavones, such as isoorientin, isovitexin, and isoscoparin, and were not anthocyanins or their derivatives (Kim et al. 2018). Flavonoid biosynthesis is controlled by structural and regulatory genes, and the genes involved in the flavonoid pathway are differentially regulated in monocot and dicot species, as indicated by the regulation patterns of regulatory genes (Petroni and Tonelli 2011), functional similarities of homologous genes (Spelt et al. 2000), and species-specific enzymes (Tohge et al. 2017). Taken together, this suggests that the biosynthetic pathways of anthocyanins and *C*-glycosyl flavones are regulated by transcription factors that interact with *COP1*, but that these transcription factors and/or their interactions with *COP1* may differ among plant species. This also suggests that the transcription factors and genes downstream of *COP1* that are involved in the flavonoid biosynthesis pathway may be differentially regulated in monocots and dicots.

In *Arabidopsis*, *cop1* mutants have been separated into three classes, weak, strong, and lethal, according to their light reactivity (McNellis et al. 1994). Homozygous *cop1* mutant seedlings in both the weak and strong categories exhibited light-grown characteristics when grown under dark conditions, albeit with a strongly reduced seed set. Homozygous lethal *cop1* mutant seedlings exhibited a similar light-grown phenotype in darkness, but also had dark purple seeds and were seedling lethal. Mutations in the *C*-terminal region of the COP1 protein, containing the WD40 repeat regions, produced a much more severe phenotype, similar to that of complete loss-of-function mutants, than mutations in the *N*-terminal region. Many *cop* mutants have mutations within the WD40 repeat region, including *cop1-5*, *cop1-7*, and *cop1-8*, all of which display a lethal phenotype (Ma et al. 2003; McNellis et al. 1994; Zhou et al. 1998). In rice, the *pps-1* mutation is located between the coiled-coil and WD40 domains, similar to weak *cop1* alleles in *Arabidopsis* such as c*op1*-*4* and *cop*1-*6*, which contain mutations in the *N*-terminal (Stacey et al. 2000). The rice *pps-1* mutation was dark-green in color and had a dwarf phenotype, contrasting with *pps*-*2*, which had a premature stop codon in the WD40 domain and exhibited ~ 5% germination (Tanaka et al. 2011). Of the two *pps* mutants, it is likely that the *pps*-*2* mutant allele, which has a mutation in the *C*-terminal of *COP1*, represents a more severe phenotype than *pps*-*1*. In this study, three different mutations in the RING finger, coiled-coil, and WD40 repeat domains of YEL caused similar phenotypic changes: embryonic lethality and accumulation of flavonoids in the pericarp and embryo. Interestingly, although the *pps*-*2* mutation was located between the mutations in the CPR_WD-23 and CPR_WD-13 knockout lines (Fig. 4a and b), which generated premature stop codons three amino acids before and eight amino acids after the *pps*-*2* mutation point, respectively, *pps-2* did not exhibit the same phenotype as the *yel* mutants, although the *pps*-*2* germination rate was extremely low and plants died within a week. These results suggest that the *yel* phenotype and other severe phenotypes can be induced by knocking out the crucial domain of *COP1* in rice, irrespective of whether the mutation is in the *N*-terminal or *C*-terminal.

Knockout of essential genes generally leads to lethality or sterility, and knockout approaches are therefore limited in their capacity to yield informative results regarding gene function when the gene plays a crucial role in the organism (Zimmer et al. 2019). However, in this study, complete gene knockout using the CRISPR/Cas9 system was preferable to knockdown with RNA interference (RNAi), as difficulties were experienced in obtaining transgenic RNAi plants with the *yel* phenotype by reducing *COP1* expression. Although several transgenic plants carrying RNAi constructs were generated, the *yel* phenotype was not observed in T_1_ or T_2_ seeds (data not shown). Attempts were also made to generate *COP1* loss-of-function plants through anther culture. However, no homozygous recessive plants were obtained from a total of 293 double haploid plants derived from anther culture (data not shown). These experiences indicated that the RNAi approach did not downregulate *COP1* expression to a sufficiently low level to induce the *yel* phenotype, and that recessive deleterious or lethal genes, such as *cop1*, might cause callus cell death, eliminating haploid plants with homozygous recessive *cop1* alleles during anther culture. However, CRISPR/Cas9 editing produced several heterozygous T_0_ transgenic plants that produced segregating *yel* phenotype seeds in the T_1_ generation. Moreover, an egg cell-specific promoter rather than the 35S promoter was used to drive Cas9 expression (Lee et al. 2019; Wang et al. 2015) to overcome potential lethality during tissue culture regeneration of T_0_ plants and to increase the frequency of the *yel* phenotype in T_1_ seeds. Subsequently, homozygous mutant seeds displaying the *yel* phenotype were observed in the T_1_ generation (Fig. 4a and b). In this study, targeted gene editing was successfully used to show that *COP1* was essential for flavonoid biosynthesis and embryo development, demonstrating the utility of CRISPR/Cas9 editing for examining previously uninducible phenotypes masked by lethality.

Previous studies have shown that the *cop1*-*5* null mutant exhibited seedling lethal and high level of anthocyanin accumulation was successfully restored by full-length *AtCOP1* and *OsCOP1* (Holm et al. 2001; Ranjan et al. 2014; Stacey et al. 2000) in *Arabidopsis*. In addition, overexpression of the COP1 (1–282) fragment restored a wild-type phenotype in the *cop1*-*5* mutant seedling in darkness (Stacey et al. 2000). However, normal pericarp color but black colored embryo seeds (partial recovery-type) were produced when the full-length Os*COP1* cDNA was overexpressed in a homozygous recessive *yel-hc* background seed (Fig. 4a and c). Even though the contents of flavonoids were significantly reduced in grain, embryo, and endosperm, the level of flavonoids in the embryo was still higher than that of wild-type (Fig. 4d–f). Moreover, the embryonic lethality was not rescued in the partially restored seeds. It is known that COP1 interacts with SUPPRESSOR OF PHYA-105 (SPA1) through their coiled-coil regions (Hoecker and Quail 2001), and the COP1/SPA complex targets transcription factors, such as ELONGATED HYPOCOTYL5 (HY5), HY5 HOMOLOG (HYH), LONG HYPOCOTYL IN FAR-RED 1 (HFR1), and PRODUCTION OF ANTHOCYANIN PIGMENT 1 and 2 (PAP1 and PAP2) involved in photomorphogenesis and anthocyanin biosynthesis for ubiquitination and degradation in *Arabidopsis* (Holm et al. 2002; Jang et al. 2005; Maier et al. 2013; Saijo et al. 2003). In particular, HY5, a bZIP transcription factor, positively regulates anthocyanin biosynthesis related structural genes through direct binding to their promoters (Shin et al. 2007). In the process of restoration, the competition between the mutated form of YEL protein (*yel*-*hc*) and normal OsCOP1/YEL to interact with SPA1 may reduce E3 ligase activity of COP1, and it may prevent ubiquitination and degradation of HY5 through the 26S proteasome pathway. Thus, remained HY5 enables to accumulate the flavonoids by inducing the expression of transcription factor in the seed. Therefore, it is assumed that the weakened E3 ligase activity of COP1 caused by the mutated COP1 that interfered the interaction between normal COP1 and SPA1 led to the incomplete COP1-mediated ubiquitination and degradation of HY5, resulting in partial restoration of *yel* phenotype in rice.

It was previously reported that expression of *OsCOP1* was observed in almost all of rice tissues, such as calli, roots, and leaves (Tsuge et al. 2001). In addition, *PPS*, an ortholog of *Arabidopsis COP1* was primarily expressed in leaves than shoot apices and young panicles during the juvenile-adult transition stage in rice; the lowest expression of *PPS* was shown in second leaves, and the expression was reached a peak in fourth and fifth leaves, but declined in higher leaves (Tanaka et al. 2011). However, unlike previous studies, negligible GUS activity of *OsCOP1* was detected in leaf in this study (Fig. 5f). It is probably because the leaf was sampled at the flowering stage, which was after finishing the juvenile-adult transition, or might be sampled at the lower leaf of plant, which had the low expression level of *OsCOP1* for GUS staining.

In this study, we identified three novel mutations responsible for the yellowish-pericarp and embryo lethal (*yel*) phenotype, and showed that *COP1*, a key repressor of light signaling, played a role in flavonoid biosynthesis and embryogenesis in rice seed. Further examination of the *YEL* locus will facilitate a better understanding of the molecular mechanisms involved in embryo development, and will provide novel insights into the roles of flavonoid biosynthesis-related genes and their interactions in rice.

## Supplementary Information

Below is the link to the electronic supplementary material.

Supplementary file1 (PDF 613 kb)
