# Supplementary material for: OsCOP1 regulates embryo development and flavonoid biosynthesis in rice (Oryza sativa L.)
Source: Theor Appl Genet. 2021 May 5;134(8):2587–601. doi: 10.1007/s00122-021-03844-9 (PMC8277627; doi:10.1007/s00122-021-03844-9)
Supplement: Supplementary file 1 — Supplementary file1 (PDF 613 kb) [file 122_2021_3844_MOESM1_ESM.pdf]

Supplementary Information

Article title:
OsCOP1 regulates embryo development and flavonoid biosynthesis in rice (*Oryza sativa* L.)

Journal:

Theoretical and applied genetics

Authors:

Backki Kim, Rihua Piao, Gileung Lee, Eunbyeol Koh, Yunjoo Lee, Sunmin Woo, Reflinur, Wenzhu Jiang, Endang M. Septiningsih, Michael J. Thomson, and Hee-Jong Koh

Name, affiliation, and email of corresponding author:

Hee-Jong Koh

Department of Plant Science, Research Institute for Agriculture and Life Sciences, and Plant Genomics and Breeding Institute, Seoul National University, Seoul 08826, Republic of Korea

E-mail: heejkoh@snu.ac.kr

Content:

Table S1. Information of the primers used in this study

Fig. S1. Longitudinal sections of wild-type (Hwacheong) and *yel-hc* mature embryos.

Fig. S2. Detection and analysis of *YEL* transcripts.

Table S2. Genotype for overexpression transgene and *YEL*/*yel* background in F_2 transgenic seeds

Table S1. Information of the primers used in this study

Oligo name	Forward primer (5’-3’)	Reverse primer (5’-3’)	Purpose	Note
S02135	GAGTGCGGAACTGTGGAAGT	GGATATCCTCCATTAAGAGAGAAGTG	Mapping	InDel
S02C	GAGCCTTCCTCCCCACTC	TATCTCTACCGGCAGCATCC	Mapping	InDel
S02F	CATGTGATCCCCAAGAATCC	TGGCCCTACTGCTAATTGCT	Mapping	InDel
S02I	CTCGGTGTCGTACAGCTTCA	TCCTGTCCGCTTCAGGTAAC	Mapping	InDel
S02K-1	GGCTCTTCTTGTCCATGAGC	GACTACTCGCCGCAGAGC	Mapping	InDel
S02K-2	GTGAGAGAATTTCAATCGGTGA	CATGCACACGTTTCGGTACT	Mapping	InDel
S02M	AACTTGAGGTCTAGCCCAGAGA	ATGTTGAGCCCACGTATCAG	Mapping	InDel
S02140	TGGGAGGAGGATATTGTGGA	TGACAGGTTGATGTGATGGAA	Mapping	InDel
yel_hc_cs	GTCTCCTCCCACCCATACAC	TGTCCACTGCTGGTGATTCT	Co-segregation analysis for yel_hc	InDel
yel_sk_cs	AAAATGTCAGCTCGCCAAAT	CAAGCACAGCTAAGGTACGG	Co-segregation analysis for yel_sk	CAPS (Mbo I)
yel_cc_cs	AATTTGACCGTGATGATGAGC	ACCCTCATAGTCGCTGCTTG	Co-segregation analysis for yel_cc	CAPS (Taq I)
COP1_RFg1	gtgtgCCACCGTCGAGTCACCCATG	aaacCATGGGTGACTCGACGGTGGc	CRISPR/Cas9 construct	
COP1_RFg2	gtgtgCGGGCAGAGGAGGTCCCTGT	aaacACAGGGACCTCCTCTGCCCGc	CRISPR/Cas9 construct	
COP1_CCg1	gtgtgTAGTCTTATGACTTTGATCG	aaacCGATCAAAGTCATAAGACTAc	CRISPR/Cas9 construct	
COP1_CCg2	gtgtgCAAAGAAGATATAAGTGCTG	aaacCAGCACTTATATCTTCTTTGc	CRISPR/Cas9 construct	
COP1_WDg1	gtgtGATGAGCTATTTGCTACTGC	aaacGCAGTAGCAAATAGCTCATC	CRISPR/Cas9 construct	
COP1_WDg2	gtgtgATGAGGGTATAGTAACTGTT	aaacAACAGTTACTATACCCTCATc	CRISPR/Cas9 construct	
OsCOP1_HC_OX	GCAATTATTCACGCCCAGTC	TGATGTTCATTTCTCAATTCTCACG	Overexpression	
OsCOP1_HC_GUS	AAAAAGCAGGCTCTGAACCCCAGTCCTCGTTA	AGAAAGCTGGGTGCGGCAGGAGGAGGCCGCCC	GUS assay	
RT_OsCOP1	GGGAGTGAAACGAATGAGGT	TCCAGCAGACTGCGCTAATA	qRT-PCR	
yel_type_G	GTCTCCTCCCACCCATACAC	TGTCCACTGCTGGTGATTCT	Transgenic seed genotyping	*yel*
WT_type_G	GCATCTCAGCCACAAGAGC	TGTCCACTGCTGGTGATTCT	Transgenic seed genotyping	*YEL*
35S_*OsCOP1*_G	CTATCCTTCGCAAACCCTTC	GGAGGAAGTTGGGGTAGAGC	Transgenic seed genotyping	35S_*OsCOP1*
HPT	GTAAATAGCTGCGCCGATGG	TACTTCTACACAGCCATCGG	Transgenic seed genotyping	HPT

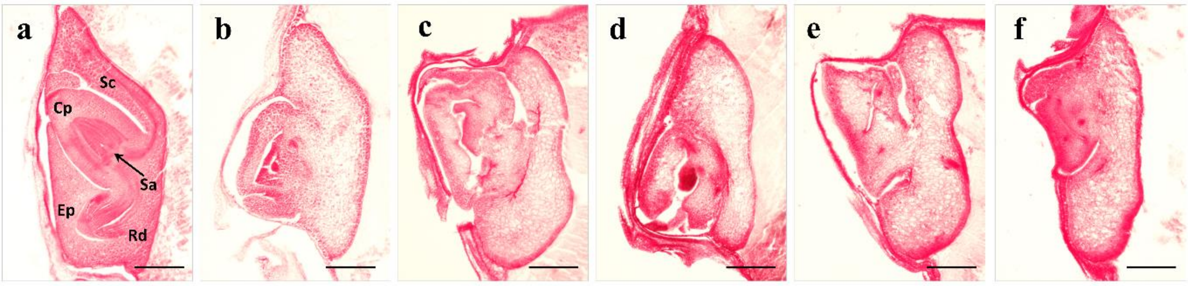

Fig. S1. Longitudinal sections of wild-type (Hwacheong) and *yel-hc* mature embryos. **a.** Mature wild-type embryo (40 DAP). **B-f.** Mature *yel-hc* mutant embryos (Bar = 300 μm). Cp, coleoptile; Ep, epiblast; Rd, radicle; Sc, scutellum; Sa, shoot apex.

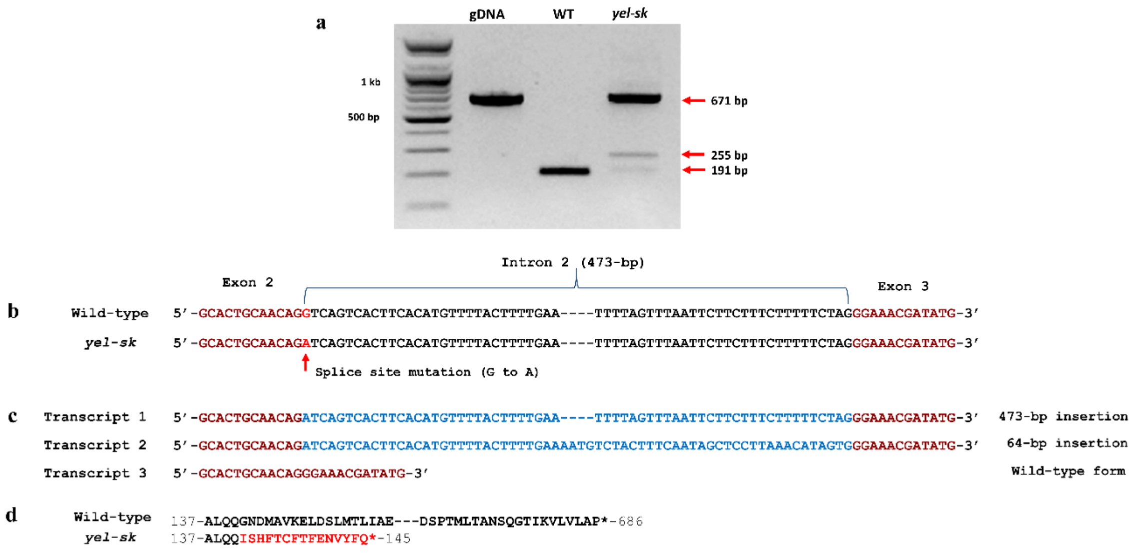

Fig. S2. Detection and analysis of *YEL* transcripts. **a.** RT-PCR analysis of *YEL* transcripts in wild-type and *yel-sk*. Numbers indicate amplicon lengths. **b.** Genomic DNA sequence and mutation point of wild type and *yel-sk*. **c.** Wild-type sequence and alternatively spliced transcript sequences in the *yel-sk* mutant. **d.** Comparison of wild-type and *yel-sk* amino acid sequences. The stop codon is indicated with an asterisk.

Table S2. Genotype for overexpression transgene and *YEL*/*yel* background in F_2 transgenic seeds

NO	Sub. No.	Phenotype of F_2 seed	Genotype				
			yel	***YEL***	**35S_*OsCOP1***	**HPT**	**Genotype of F_2 seed**
1	YEL_OX-1-Y-1	yel mutant-type	+	-	-	-	yel/yel
2	YEL_OX-1-Y-2	yel mutant-type	+	-	-	-	yel/yel
3	YEL_OX-1-Y-3	yel mutant-type	+	-	-	-	yel/yel
4	YEL_OX-1-Y-4	yel mutant-type	+	-	-	-	yel/yel
5	YEL_OX-1-Y-5	yel mutant-type	+	-	-	-	yel/yel
6	YEL_OX-1-Y-6	yel mutant-type	+	-	-	-	yel/yel
7	YEL_OX-3-Y-1	yel mutant-type	+	-	-	-	yel/yel
8	YEL_OX-3-Y-2	yel mutant-type	+	-	-	-	yel/yel
9	YEL_OX-3-Y-3	yel mutant-type	+	-	-	-	yel/yel
10	YEL_OX-3-Y-4	yel mutant-type	+	-	-	-	yel/yel
11	YEL_OX-4-Y-1	yel mutant-type	+	-	-	-	yel/yel
12	YEL_OX-4-Y-2	yel mutant-type	+	-	-	-	yel/yel
13	YEL_OX-5-Y-1	yel mutant-type	+	-	-	-	yel/yel
14	YEL_OX-5-Y-2	yel mutant-type	+	-	-	-	yel/yel
15	YEL_OX-5-Y-3	yel mutant-type	+	-	-	-	yel/yel
16	YEL_OX-6-Y-1	yel mutant-type	+	-	-	-	yel/yel
17	YEL_OX-6-Y-2	yel mutant-type	+	-	-	-	yel/yel
18	YEL_OX-6-Y-3	yel mutant-type	+	-	-	-	yel/yel
19	YEL_OX-8-Y-1	yel mutant-type	+	-	-	-	yel/yel
20	YEL_OX-8-Y-2	yel mutant-type	+	-	-	-	yel/yel
21	YEL_OX-1-P-1	Partial recovery-type	+	-	+	+	yel/yel + 35S_OsCOP1
22	YEL_OX-1-P-2	Partial recovery-type	+	-	+	+	yel/yel + 35S_OsCOP1
23	YEL_OX-1-P-3	Partial recovery-type	+	-	+	+	yel/yel + 35S_OsCOP1
24	YEL_OX-1-P-4	Partial recovery-type	+	-	+	+	yel/yel + 35S_OsCOP1
25	YEL_OX-1-P-5	Partial recovery-type	+	-	+	+	yel/yel + 35S_OsCOP1
26	YEL_OX-3-P-1	Partial recovery-type	+	-	+	+	yel/yel + 35S_OsCOP1
27	YEL_OX-3-P-2	Partial recovery-type	+	-	+	+	yel/yel + 35S_OsCOP1
28	YEL_OX-3-P-3	Partial recovery-type	+	-	+	+	yel/yel + 35S_OsCOP1
29	YEL_OX-3-P-4	Partial recovery-type	+	-	+	+	yel/yel + 35S_OsCOP1
30	YEL_OX-5-P-1	Partial recovery-type	+	-	+	+	yel/yel + 35S_OsCOP1
31	YEL_OX-5-P-2	Partial recovery-type	+	-	+	+	yel/yel + 35S_OsCOP1
32	YEL_OX-5-P-3	Partial recovery-type	+	-	+	+	yel/yel + 35S_OsCOP1
33	YEL_OX-5-P-4	Partial recovery-type	+	-	+	+	yel/yel + 35S_OsCOP1
34	YEL_OX-5-P-5	Partial recovery-type	+	-	+	+	yel/yel + 35S_OsCOP1

35	YEL_OX-6-P-1	Partial recovery-type	+	-	+	+	yel/yel + 35S_OsCOP1
36	YEL_OX-6-P-2	Partial recovery-type	+	-	+	+	yel/yel + 35S_OsCOP1L
37	YEL_OX-8-P-1	Partial recovery-type	+	-	+	+	yel/yel + 35S_OsCOP1
38	YEL_OX-8-P-2	Partial recovery-type	+	-	+	+	yel/yel + 35S_OsCOP1
39	YEL_OX-8-P-3	Partial recovery-type	+	-	+	+	yel/yel + 35S_OsCOP1
40	YEL_OX-8-P-4	Partial recovery-type	+	-	+	+	yel/yel + 35S_OsCOP1
41	YEL_OX-1-N-1	Normal-type	-	+	+	+	YEL/YEL + 35S_OsCOP1
42	YEL_OX-1-N-2	Normal-type	-	+	+	+	YEL/YEL + 35S_OsCOP1
43	YEL_OX-1-N-3	Normal-type	+	+	+	+	YEL/yel + 35S_OsCOP1
44	YEL_OX-1-N-4	Normal-type	+	+	+	+	YEL/yel + 35S_OsCOP1
45	YEL_OX-1-N-5	Normal-type	-	+	-	-	YEL/YEL
46	YEL_OX-1-N-6	Normal-type	+	+	+	+	YEL/yel + 35S_OsCOP1
47	YEL_OX-1-N-7	Normal-type	+	+	+	+	YEL/yel + 35S_OsCOP1
48	YEL_OX-1-N-8	Normal-type	+	+	+	+	YEL/yel + 35S_OsCOP1
49	YEL_OX-1-N-9	Normal-type	+	+	-	-	YEL/yel
50	YEL_OX-1-N-10	Normal-type	+	+	+	+	YEL/yel + 35S_OsCOP1
51	YEL_OX-3-N-1	Normal-type	-	+	+	+	YEL/YEL + 35S_OsCOP1
52	YEL_OX-3-N-2	Normal-type	+	+	-	-	YEL/yel
53	YEL_OX-3-N-3	Normal-type	+	+	+	+	YEL/yel + 35S_OsCOP1
54	YEL_OX-3-N-4	Normal-type	+	+	+	+	YEL/yel + 35S_OsCOP1
55	YEL_OX-3-N-5	Normal-type	-	+	-	-	YEL/YEL
56	YEL_OX-3-N-6	Normal-type	-	+	+	+	YEL/YEL + 35S_OsCOP1
57	YEL_OX-3-N-7	Normal-type	+	+	+	+	YEL/yel + 35S_OsCOP1
58	YEL_OX-3-N-8	Normal-type	+	+	-	-	YEL/yel
59	YEL_OX-3-N-9	Normal-type	+	+	+	+	YEL/yel + 35S_OsCOP1
60	YEL_OX-3-N-10	Normal-type	+	+	+	+	YEL/yel + 35S_OsCOP1
61	YEL_OX-5-N-1	Normal-type	+	+	+	+	YEL/yel + 35S_OsCOP1
62	YEL_OX-5-N-2	Normal-type	+	+	+	+	YEL/yel + 35S_OsCOP1
63	YEL_OX-5-N-3	Normal-type	-	+	+	+	YEL/YEL + 35S_OsCOP1
64	YEL_OX-5-N-4	Normal-type	-	+	+	+	YEL/YEL + 35S_OsCOP1
65	YEL_OX-5-N-5	Normal-type	-	+	+	+	YEL/YEL + 35S_OsCOP1
66	YEL_OX-5-N-6	Normal-type	+	+	+	+	YEL/yel + 35S_OsCOP1
67	YEL_OX-5-N-7	Normal-type	+	+	-	-	YEL/yel
68	YEL_OX-5-N-8	Normal-type	-	+	+	+	YEL/YEL + 35S_OsCOP1
69	YEL_OX-5-N-9	Normal-type	+	+	+	+	YEL/yel + 35S_OsCOP1
70	YEL_OX-5-N-10	Normal-type	-	+	+	+	YEL/YEL + 35S_OsCOP1
71	YEL_OX-6-N-1	Normal-type	-	+	+	+	YEL/YEL + 35S_OsCOP1

72	YEL_OX-6-N-2	Normal-type	+	+	+	+	YEL/yel + 35S_OsCOP1
73	YEL_OX-6-N-3	Normal-type	+	+	+	+	YEL/yel + 35S_OsCOP1
74	YEL_OX-6-N-4	Normal-type	+	+	+	+	YEL/yel + 35S_OsCOP1
75	YEL_OX-6-N-5	Normal-type	+	+	+	+	YEL/yel + 35S_OsCOP1
76	YEL_OX-6-N-6	Normal-type	+	+	-	-	YEL/yel
77	YEL_OX-6-N-7	Normal-type	+	+	-	-	YEL/yel
78	YEL_OX-6-N-8	Normal-type	-	+	+	+	YEL/YEL + 35S_OsCOP1
79	YEL_OX-6-N-9	Normal-type	-	+	-	-	YEL/YEL
80	YEL_OX-6-N-10	Normal-type	-	+	-	-	YEL/YEL
81	YEL_OX-8-N-1	Normal-type	+	+	-	-	YEL/yel
82	YEL_OX-8-N-2	Normal-type	+	+	-	-	YEL/yel
83	YEL_OX-8-N-3	Normal-type	+	+	+	+	YEL/yel + 35S_OsCOP1
84	YEL_OX-8-N-4	Normal-type	+	+	+	+	YEL/yel + 35S_OsCOP1
85	YEL_OX-8-N-5	Normal-type	+	+	+	+	YEL/yel + 35S_OsCOP1
86	YEL_OX-8-N-6	Normal-type	+	+	-	-	YEL/yel
87	YEL_OX-8-N-7	Normal-type	+	+	+	+	YEL/yel + 35S_OsCOP1
88	YEL_OX-8-N-8	Normal-type	+	+	-	-	YEL/YEL + 35S_OsCOP1
89	YEL_OX-8-N-9	Normal-type	+	+	-	-	YEL/yel
90	YEL_OX-8-N-10	Normal-type	+	+	+	+	YEL/yel + 35S_OsCOP1
91	YEL_OX-8-N-11	Normal-type	+	+	-	-	YEL/yel
92	YEL_OX-8-N-12	Normal-type	-	+	-	-	YEL/YEL
93	YEL_OX-8-N-13	Normal-type	-	+	-	-	YEL/YEL

yel: *yel-hc* allele (mutant), *YEL*: *YEL* allele (wild-type), 35S_*OsCOP1*: Overexpression transgene, HPT: hygromycin B selection
